# Development of a rapid, sensitive, and selective LC–MS/MS method for quantifying curcumin levels in healthy human urine: Effect of pepper on curcumin bioavailability

**DOI:** 10.1002/fsn3.3691

**Published:** 2023-09-18

**Authors:** Sana Khajeh pour, Cynthia Blanton, Biwash Ghimire, Ali Aghazadeh‐Habashi

**Affiliations:** ^1^ Department of Biomedical and Pharmaceutical Sciences Idaho State University Pocatello Idaho USA; ^2^ Department of Nutrition and Dietetics Idaho State University Pocatello Idaho USA

**Keywords:** bioavailability, curcumin, LC–MS/MS, piperine, urinary excretion

## Abstract

Curcumin (CCM), a culinary spice, is widely consumed for its health benefits for managing oxidative and inflammatory conditions, metabolic syndrome, arthritis, and hyperlipidemia. However, due to its extensive metabolism, the oral bioavailability of CCM is very low. In this study, we developed a rapid, sensitive, and selective assay to examine the hypothesis that piperine improves CCM bioavailability after piperine co‐ingestion. We developed a selective, sensitive, and robust LC–MS/MS method to quantify CCM in human urine. The method was linear over a concentration range 0.625–40 ng/mL with LLOQ and LLOD of 0.625 ng/mL and 0.312 ng/mL, respectively. Healthy volunteers have consumed test meals of CCM as turmeric powder with and without black pepper with 1 week wash out. Urine samples were collected for 24 hours and analyzed for CCM excretion. Black pepper increased CCM half‐life from 2.2 ± 0.79 h (CCM alone) to 4.5 ± 0.80 h (CCM + pepper). The CCM 24‐h urinary excreted amount was higher in individuals consuming CCM + pepper (218.14 ± 94.98 μg) than those who received CCM only (49.45 ± 12.94 μg). This preliminary study indicates that piperine significantly increased CCM oral absorption, reduced systemic clearance, and improved bioavailability.

## Graphical abstract

Due to its extensive metabolism, the oral bioavailability of CCM is very low. The administration of CCM with piperine improved bioavailability by increasing its oral absorption and reducing systemic clearance.

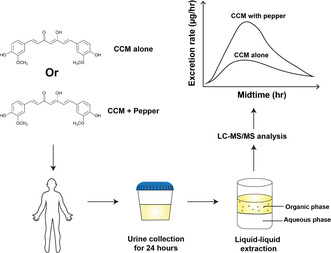

## 1 INTRODUCTION

Spice consumption confers protection against inflammation, oxidative damage, neurodegeneration, metaplasia, and their resulting diseases (Bengmark, 2006; Kaefer & Milner, 2008; Lai & Roy, 2004; Opara & Chohan, 2014; Pandey & Rizvi, 2009; Tsai et al., 2007). Curcumin (CCM), a polyphenolic pigment in turmeric, interacts with cellular and molecular targets to enhance the expression of tumor suppressor genes and suppress inflammatory signaling cascades (Aggarwal & Sung, 2009; Chainani‐Wu, 2003; Kunnumakkara et al., 2008). However, research on the health benefits of CCM is hampered by the difficulty in assessing its intake and absorption extent in humans. Spices are consumed in small amounts sporadically, rendering intake measurement imprecise, and the bioactive components of spices, including turmeric, are minimally bioavailable (Anand et al., 2007; Opara & Chohan, 2014). On the other hand, the possible side effects of a natural product can be an issue if it is not used in the proper amount. CCM has an established safety profile based on the allowable daily intake of up to 3 mg/kg body weight (Kocaadam & Şanlier, 2017). However, there are reports of side effects such as diarrhea, headache, rash, and yellow stool and elevation of serum alkaline phosphates and lactate dehydrogenase when used in higher amounts (Anand et al., 2007; Bengmark, 2006; Lao et al., 2006; Sharma et al., 2004). It seems that the reason for the safety and lack of hepatotoxicity of CCM could be its low oral bioavailability. Different approaches, such as black pepper coadministration or nanoparticle delivery methods, have increased its absorption and bioavailability. Subsequently, the incidence of these side effects and hepatotoxicity have been reported (Anonymous, 2012; Lombardi et al., 2021; Sohal et al., 2021). Therefore, accurate and sensitive detection of turmeric exposure is a research priority for linking CCM intake to health‐beneficial outcomes and adverse effects. However, studies testing this relationship have primarily utilized CCM supplements and extracts (Tabrizi et al., 2019), which poses at least two limitations: (1) CCM supplements do not represent the most common route of CCM intake, which is culinary turmeric consumption; and (2) extrapolating biomarker response from experimental CCM supplement administration to real‐life CCM supplement use does not account for the variability in purity in the supplement marketplace (Ichim & Booker, 2021; Skiba et al., 2018). This is an essential consideration since large observational studies that contribute significantly to the evidence‐based relating dietary intake to health outcomes rely on biomarkers of nutrient intakes from food (e.g., culinary turmeric) rather than supplements (e.g., CCM supplements of variable potencies) (Imamura et al., 2018; Zheng et al., 2020). Thus, the need exists to develop a method of CCM biomarker detection in humans consuming turmeric at a typical dose from food. Dietary biomarkers are valuable for assessing individual nutrient and food intakes and their interactions. It is well documented that the bioavailability of polyphenols such as CCM is limited. Animal studies indicate poor systemic bioavailability of CCM stemming from rapid metabolic reduction and conjugation after oral intake (Asai & Miyazawa, 2000; Ireson et al., 2001; Pan et al., 1999). For example, oral administration of 0.1 g/kg of CCM in mice resulted in only 2.25 μg/mL peak plasma concentration (Pan et al., 1999). After oral administration of 500 mg/kg of CCM in rats, the peak plasma concentration was 1.8 ng/mL (Ireson et al., 2001). Five hundred mg oral administration of CCM in rats provided a bioavailability of only 1% (Yang et al., 2007). Similarly, low bioavailability of CCM was found in humans receiving 8 g CCM daily for 8 weeks. Plasma steady‐state levels of CCM on day 3 in these patients reached only 22–41 ng/mL (Dhillon et al., 2008). The co‐ingestion with black pepper significantly improves the bioavailability of CCM.

Piperine, a bioactive compound in black pepper, achieves this by inhibiting CCM hepatic and intestinal metabolism (Panahi et al., 2015; Shoba et al., 1998; Volak et al., 2008). Pharmacokinetic interactions between food components determine diet–health relationships (Moughan, 2020). Identifying the magnitude of the interaction effect, be it synergism, addition, or antagonism, in a mixed meal, such as turmeric and black pepper, is another critical factor in interpreting biomarker data related to health outcomes (Wang et al., 2011; Zhang et al., 2019). Thus, a need exists for a robust method to measure CCM levels in humans receiving turmeric at a typical dose from food sources. There are several conventional analytical methods in the literature that do not provide enough sensitivity to detect CCM in biological matrices. However, the availability of the LC–MS/MS technique provides an indispensable tool for various applications, including drug discovery (Jia et al., 2017), pharmacokinetic studies (Alrabiah et al., 2019; Gopi et al., 2017), bioavailability (Jude et al., 2018), clinical trials (Kunati et al., 2018), and metabolic stability assessment of different drugs (Amer et al., 2017; Kadi et al., 2016). Its combination of sensitivity, selectivity, and versatility makes it a preferred choice for researchers and analysts in the pharmaceutical and clinical industries.

Several analytical methods have been reported for quantifying CCM in plasma, urine, and tissues, but their application is limited by their lack of sensitivity (Amer et al., 2017; Bhuket et al., 2016; Heath et al., 2003; Kim et al., 2017; Li et al., 2011; Ma et al., 2015; Vareed et al., 2008). In some cases, the extraction and detection methods were unsuitable for quantifying the CCM because its concentrations were lower than other methods' limit of quantification (LLOQ) (Kunati et al., 2018). In this context, the quantification of CCM in biofluids would benefit from a straightforward, sensitive, and reliable analytical method. Therefore, we developed a simple and sensitive LC–MS/MS method to study the pharmacokinetics interaction of piperine and its impact on CCM levels in healthy individuals after ingesting a moderate dose of turmeric alone or in combination with black pepper. The urine sample was selected as the biofluid due to its noninvasive collection method.

## 2 MATERIALS AND METHODS

### 2.1 Materials and reagents

CCM synthetic (molecular weight: 368.39 g/mol) and glibenclamide (molecular weight 494.00 g/mol) as internal standard (IS) were obtained from Tokyo Chemical Industry. 3D‐deionized (DI) water was obtained from bioWORLD. Ethyl acetate was obtained from Fisher Scientific. Formic acid, methanol, HPLC‐grade methanol, HPLC‐grade water, and HPLC‐grade acetonitrile were obtained from Sigma‐Aldrich Co.

### 2.2 LC–MS/MS system

HPLC separation was performed using a Shimadzu LC system. The system is composed of an autosampler (SIL‐30 AC), high‐pressure quaternary pumps (LC‐30 AD), a column oven, the system controller (CBM‐20A), and a reverse‐phase column from InfinityLab Poroshell HPH‐C18, 4.6 × 150 mm, 2.7 μm column (Agilent Technologies). Analyte detection was performed using an AB SCIEX QTRAP 5500 quadrupole tandem mass spectrometer equipped with electrospray ionization (ESI) probe. The LC–MS/MS system was controlled by AB Sciex Analyst software (version 1.6.3) for its operation, acquisition, and processing.

### 2.3 Preparation of analytical standards, quality control, and subject samples

The stock solutions of CCM (1 mg/mL) and IS (20 ng/mL) were prepared in methanol and stored at −20°C. The standard solutions of CCM with concentrations of 0.625, 1.25, 2.5, 5, 10, 20, and 40 ng/mL were prepared by serial dilution and adding 50 μL of IS (20 ng/mL). These concentrations were made fresh for each run by spiking blank urine provided by a subject not taking any medications.

CCM analysis in each standard solution sample was done in triplicate. The 50 μL of IS stock solution was added to the urine sample aliquot (400 μL). After equilibration, 160 μL of 3D‐DI water was added and vortex mixed for 20 seconds. Then, 500 μL of the organic extraction solvent mixture (ethyl acetate: methanol 95: 5 v/v) was added to each sample, vortexed for 30 seconds, centrifuged (13,000 rpm, 4°C, 5 min), and the supernatant organic phase was collected. The extraction process was repeated twice, and the organic phases were mixed and dried using a Savant SpeedVac System (Fisher Scientific). The dried residue was then reconstituted in 50 μL acetonitrile: methanol: water: formic acid (41: 23: 36: 0.1 v/v). Aliquots of 10 μL were injected into LC–MS/MS and analyzed by the method described below.

### 2.4 LC–MS/MS analytical method

The mobile phases comprised A (0.1% formic acid in water) and B (0.1% formic acid in acetonitrile) with a 0.5 mL/min flow rate. The gradient program was used at 5% B for 0.5 min, followed by an increase to 95% in 1 minute, and held for 4.5 min. The run time was set at 8 min. The column was re‐equilibrated by decreasing the mobile phase B to 5% and maintaining it at 5% for 2 min.

Mass spectrometric quantification was carried out in the multiple reaction monitoring (MRM) mode, monitoring ion transitions of *m/z* 367.0 → 173.0 for CCM and *m/z* 494.0 → 171.8 for the IS with a collision energy of −30 eV, entrance potential of −7 eV, declustering potential of −100 eV, and collision cell exit potential of −31 eV in negative‐ion mode. The APCI source operating parameters were set as curtain gas flow of 20 psi, ion source gas flow of 30 psi, source temperature of 450°C, and an ion spray voltage of −4.5 kV.

### 2.5 Quantitative analysis, method validation, assay linearity, LLOD, and LLOQ

Standard solutions for the calibration curve were prepared in a concentration range of 0.625–40 ng/mL. The observed peak height ratios (CCM/IS) were plotted versus concentration to construct a calibration curve. The accuracy was calculated by measuring CCM in standard solutions of 0.625, 1.25, 2.5, 5, 10, 20, and 40 ng/mL in urine samples prepared as described above. The observed concentration was determined and compared with the added concentration for each sample. For determining precision, intra‐ and interday variances were defined over 1 day, 3 days, and 5 days. Three standard concentrations of 0.625, 5, and 40 ng/mL of CCM were used, and CV% was calculated for each sample analyzed. Recoveries were determined by spiking blank urine with low (0.625 ng/mL), mid (5 ng/mL), and high (40 ng/mL) concentrations of CCM, and extraction ratios were calculated. Method linearity was determined by analyzing a series of standard samples with serial dilutions on 3 consecutive days. The LLOD and LLOQ were defined as the signal‐to‐noise ratio higher than or equal to 3 and 10, respectively.

### 2.6 Dosage information for the clinical study

The clinical study was approved by the Idaho State University Human Subjects Committee (protocol IRB‐FY2021‐7), and all participants were provided with informed consent. The interindividual variability can influence the absorption, distribution, metabolism, and elimination of xenobiotics, including curcumin. To avoid and limit such variability, we enrolled healthy volunteers with normal kidney function based on their creatinine level (*n* = 3) in a crossover study in which they consumed a test meal on two occasions, separated by 1 week of washout. Individuals had free access to water. Occasion 1 meal consisted of 120 mL egg white (cooked) mixed with 5 g turmeric powder (McCormick & Co. Inc) with 2.46% of curcumin content (Amer et al., 2017). Black pepper contained a piperine concentration range 2531–8073 mg/100 g, with a mean concentration of 4418 ± 946 mg/100 g (Lee et al., 2021). Occasion 2 meal contained the same ingredients plus 0.5 g of ground black pepper (McCormick & Company Inc). Participants collected batches of urine at baseline and 10 subsequent time points for 24 h. After samples were obtained, their volume was measured, and an aliquot of them was promptly placed in a −80°C freezer and protected from light as much as possible when handling. Using the LC–MS/MS method, we determined the urine concentration of CCM and calculated the excreted amount at each interval. The urinary excretion rate (ng. h^−1^) versus mid‐time (h) graph was constructed using non‐compartmental analysis (WinNonlin version 8.3, Pharsight, A Certara Company).

### 2.7 Statistical methods

All the data are expressed as mean ± standard deviation. The student t‐test was evaluated using GraphPad Prism® statistical software program version 8.3.0 (GraphPad Prism Software Inc.). The level of significance was set at *p* < .05.

## 3 RESULTS

### 3.1 Calibration curve

**FIGURE 1:**
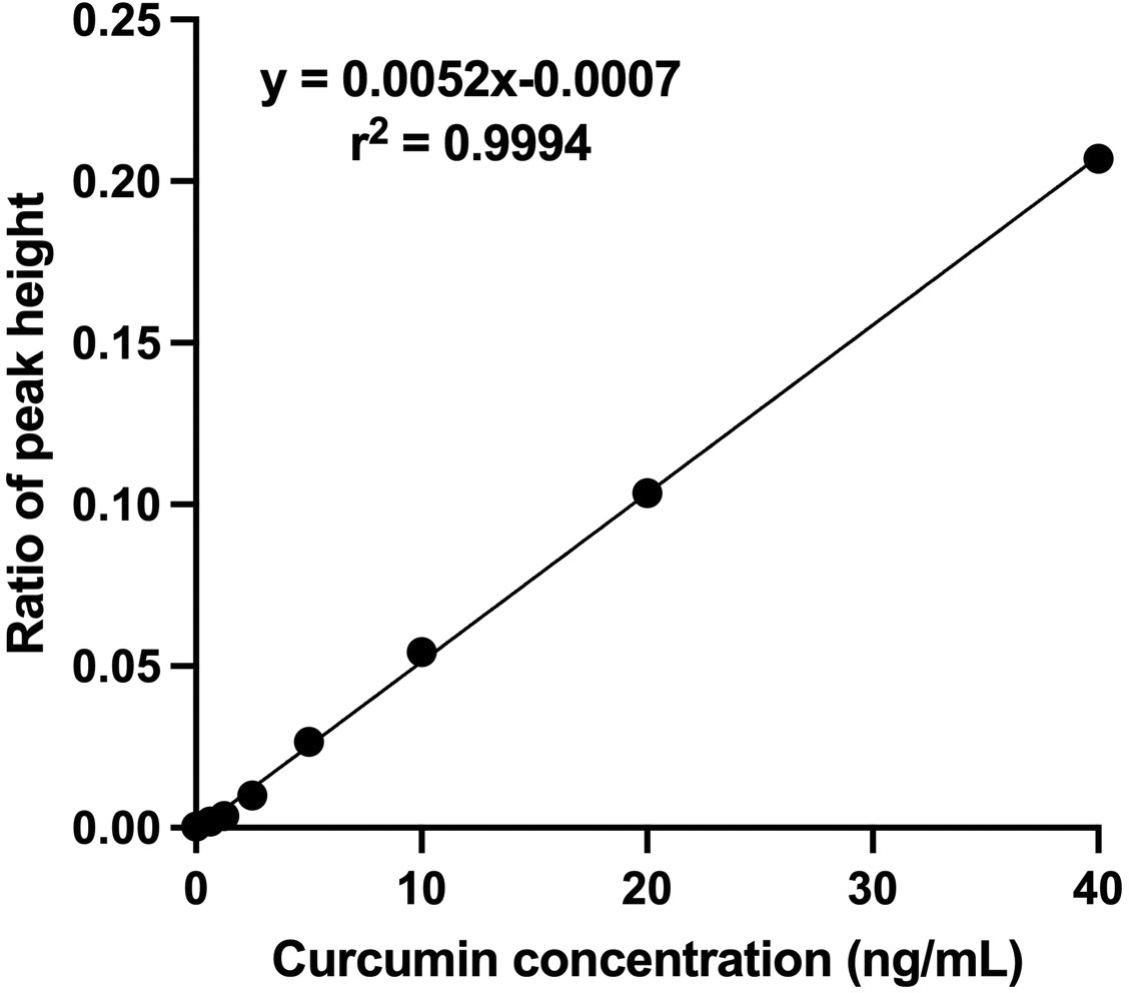
The calibration curve of CCM indicates a linear range of concentration from 0.625 to 40 ng/mL.

The calibration curve for CCM was constructed in the range 0.625–40 ng/mL, and the response was linear for the analytes throughout the concentration range (*r* ^2^ = .9994) (Figure 1). LLOQ and LLOD were 0.625 ng/mL and 0.312 ng/mL, respectively.

### 3.2 Detection of CCM in urine samples

**FIGURE 2:**
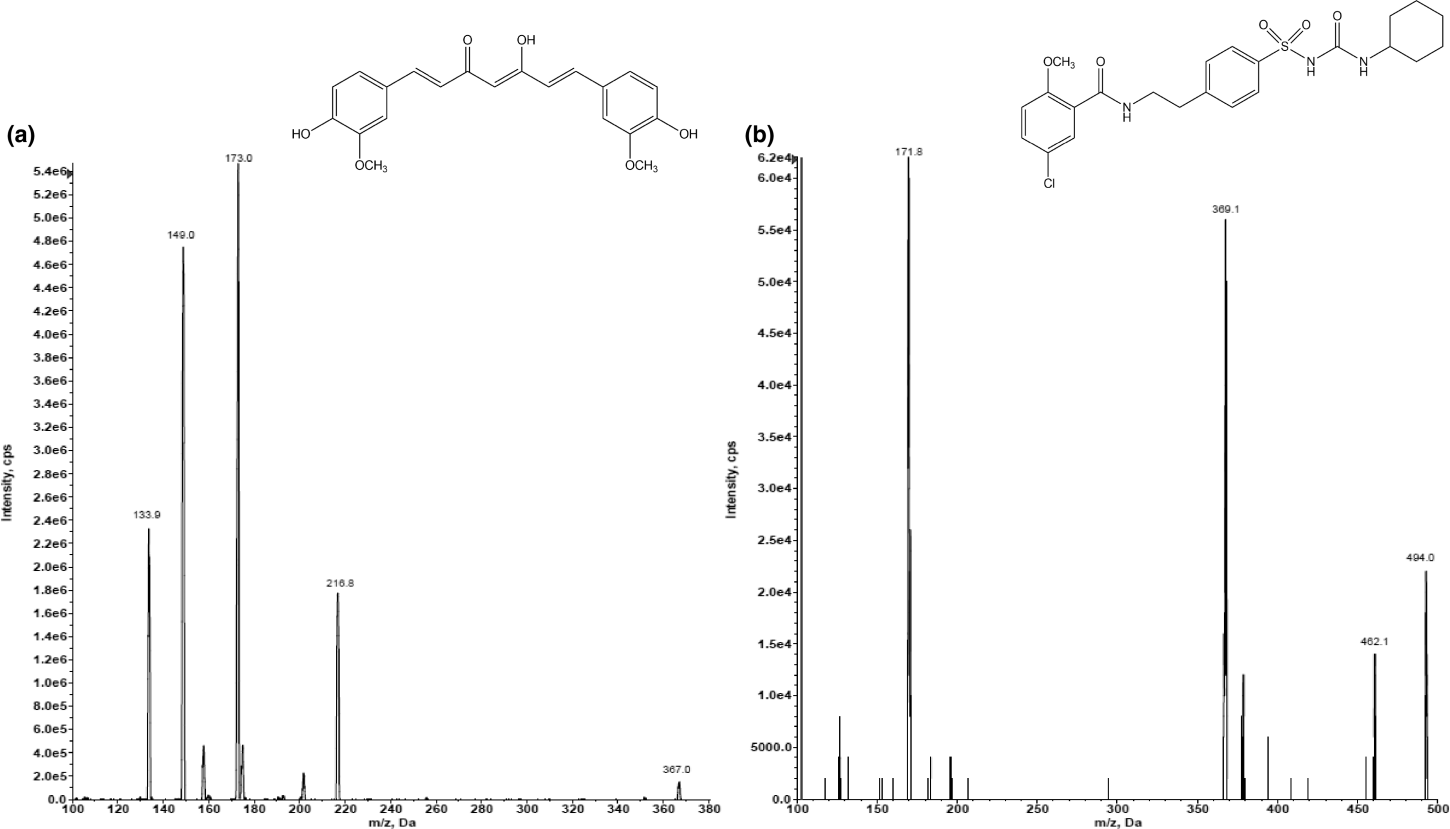
Mass spectra of CCM (a) and IS (b).

**FIGURE 3:**
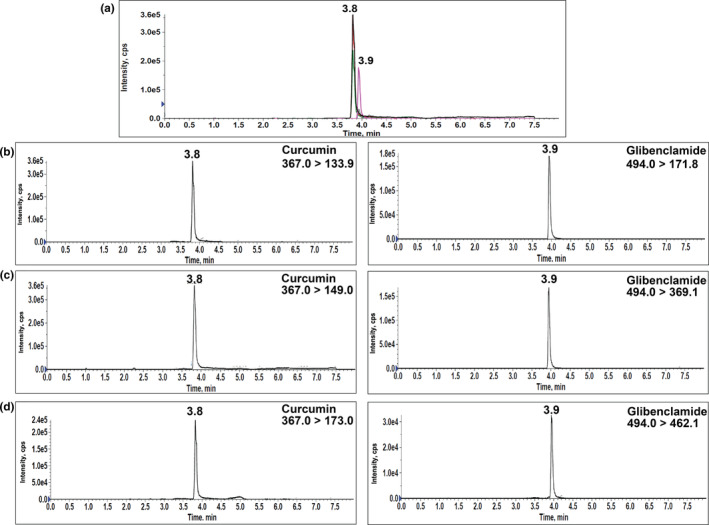
Representative MRM chromatogram of CCM and IS with different transitions. (a) Total ion chromatograms of urine‐spiked urine sample and IS. (b), (c), and (d) are different transitions tested in analysis with (a) being the major transition. CCM, curcumin; IS, internal standard; MRM, multiple reaction monitoring.

**FIGURE 4:**
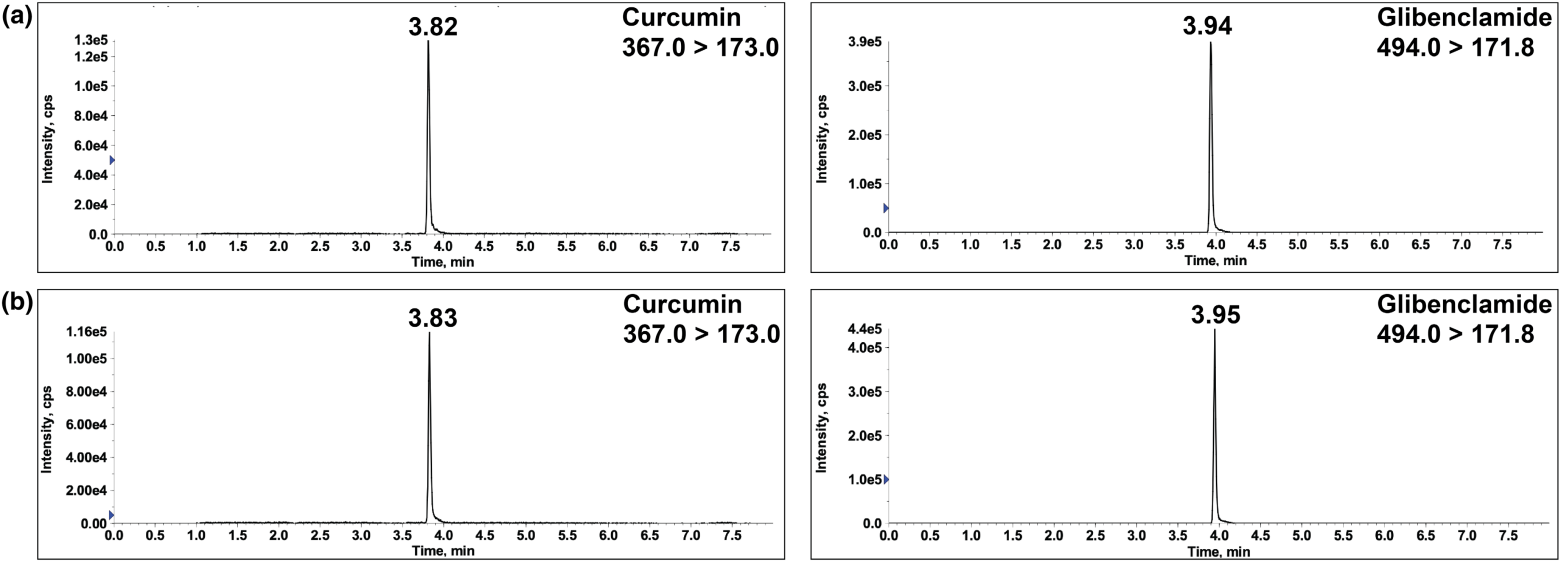
Representative MRM chromatograms of CCM and IS (a) in standard solution (10 ng/mL) and (b) spiked urine sample (10 ng/mL). CCM, curcumin; IS, internal standard; MRM, multiple reaction monitoring.

**FIGURE 5:**
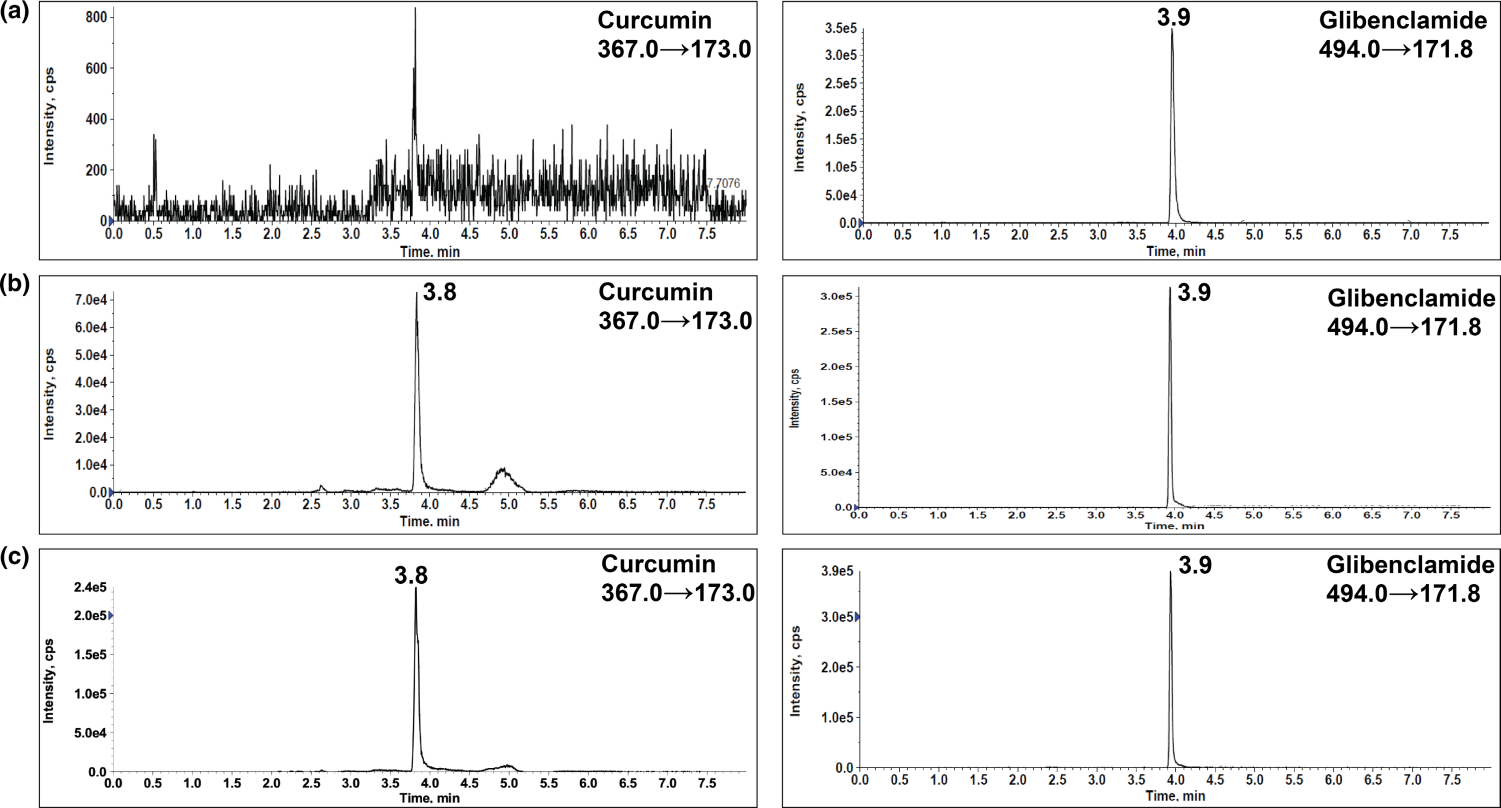
Representative MRM chromatograms of (a) blank urine containing IS, (b) urine sample of a patient in CCM only group, and (c) urine sample of the same patient in CCM + pepper group. CCM, curcumin; IS, internal standard; MRM, multiple reaction monitoring.

The fast and straightforward method described in this paper provides a reliable means for accurate and precise measurement of the urinary excretion of CCM. The mass spectra of CCM and IS can be observed in Figure 2. Three MRM transitions were used to detect CCM and IS (Figure 3) in samples and *m/z* 367.0 → 173.0 for CCM and *m/z* 494.0 → 171.8 for the IS were chosen for the analysis as the major transitions. The observed retention times for CCM and IS were approximately 3.8 and 3.9 min, respectively, in the chromatograms of standard solutions (Figure 4a), spiked urine samples (Figure 4b), and human subject urine samples (Figure 5). The lack of autosampler carryover was confirmed by injecting a blank urine sample (Figure 5a).

### 3.3 Quantitative analysis and method validation

The LC method was validated for specificity, linearity, accuracy, and intra‐day and inter‐day variations. The standard solutions were prepared in methanol by serial diluting the CCM stock solutions (1 mg/mL) and yielded samples containing 0.625, 1.25, 2.5, 5, 10, 20, and 40 ng/mL. The concentration of the IS solution was 20 ng/mL. The standard curves were constructed by plotting the analyte/IS peak height ratio versus analyte concentration. Three calibration curves were constructed on the same or 3 different days to determine intra‐ and inter‐day variabilities. The accuracy was determined from “observed concentration ×100 / added concentration” and the coefficient of variation (CV%) was used to estimate the assay precision (Table 1).

**TABLE 1:** Inter‐ and intra‐day precision and accuracy of CCM analysis in LC–MS/MS.

Concentration (ng/mL)	Intra‐day		Inter‐day	
	CV (%)	Accuracy (%) ± SD	CV (%)	Accuracy (%) ± SD
0.625	≤ 2.37	93.71 ± 2.9	≤ 9.56	84.52 ± 10.6
5	≤ 8.16	103.68 ± 12.5	≤ 5.25	93.69 ± 10.9
40	≤ 0.78	100.58 ± 1.1	≤ 0.93	98.72 ± 0.8

The recovery of CCM from spiked urine samples at low, mid, and high concentration levels were 87.2%, 95.1%, and 101.3%, respectively. Based on previous studies, the stability of CCM is minimally affected by thawing process (Kroon et al., 2023) and this is also confirmed in our study where the stability of QC samples of spiked urine and standard solutions of 5 ng/mL prepared from the 1 mg/mL stock solution was 97% which shows no significant loss over the course of analysis.

### 3.4 Pharmacokinetic and urinary excretion ratio of CCM

**FIGURE 6:**
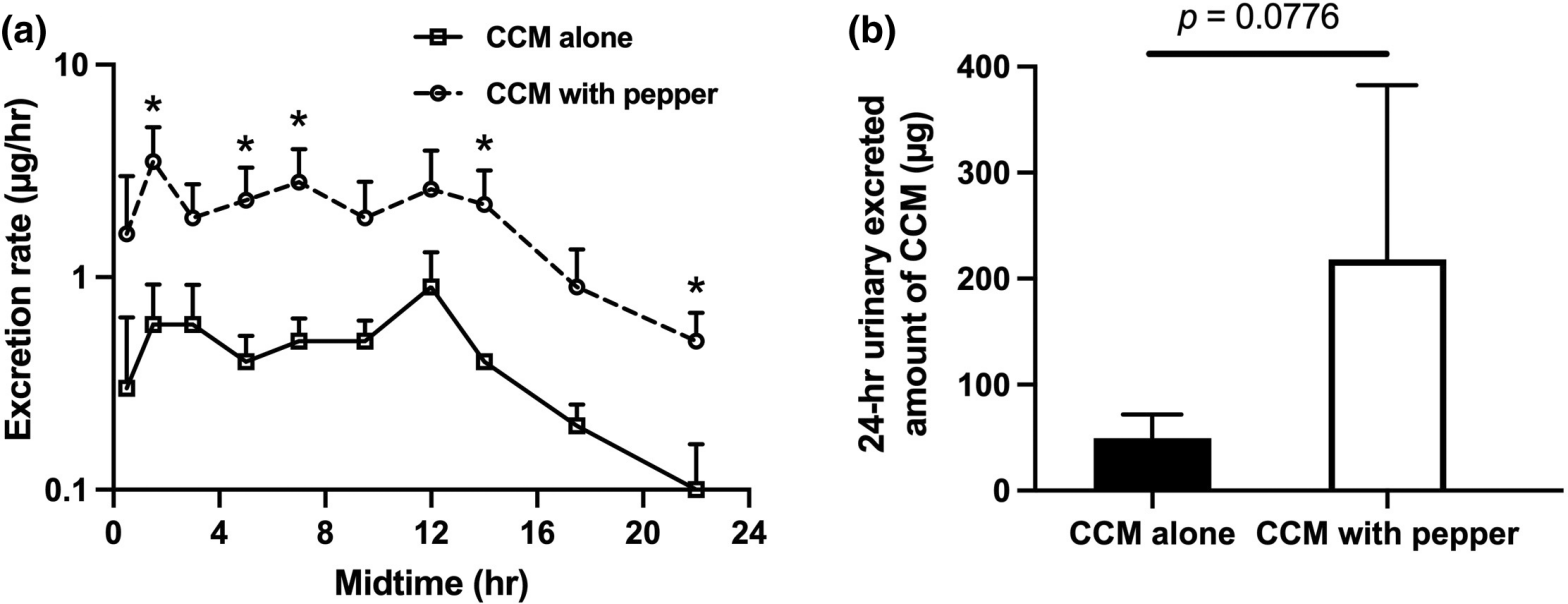
The urinary excretion rate of CCM after consumption of CCM alone or with black pepper (a) and the total excreted amount of CCM (μg/24 h) in 24 h (b). *Significantly different *p* < .05.

Urine samples of all individuals were extracted and analyzed using liquid–liquid extraction due to the hydrophobic nature of the CCM. Figure 6a shows a significantly higher excretion rate at almost all time points in individuals receiving CCM combined with black pepper. Co‐ingestion of pepper increased CCM bioavailability and half‐life by reducing its metabolic clearance. The half‐life of CCM was 2.2 ± 0.79 and 4.5 ± 0.80 h in CCM alone or with pepper groups, respectively. The total amount of CCM excreted in urine after 24 h in both groups is shown in Figure 6b. The 24‐h urinary excreted amount of CCM was higher in individuals consuming CCM with pepper (218.14 ± 94.98 μg) than in those who only received CCM (49.45 ± 12.94 μg).

## 4 DISCUSSION

The developed method in the current study for quantifying CCM in human urine is a fast, simple, sensitive, and selective method. It has significantly improved LLOD and LLOQ, with a simple liquid–liquid extraction procedure and short run time that makes it more favorable in application to biomedical and nutrition research than the previously reported methods. It has been reported that CCM has a negligible distribution to peripheral tissues (Sharma et al., 2007); therefore, its volume of distribution is less likely to be impacted by black pepper co‐ingestion. The presented comparative pharmacokinetics data, including the half‐life, CCM urinary excretion rate, and total amount execrated 24 h after oral intake of CCM alone or co‐ingestion with black pepper, indicate that black pepper significantly increases the CCM oral absorption, reduces its systemic clearance, and consequently increases its bioavailability by more than fourfold. These findings are in concert with the previous studies (Panahi et al., 2015; Shoba et al., 1998; Volak et al., 2008).

Published reports indicate that CCM has poor oral bioavailability, especially at low doses (Anand et al., 2007). CCM must be consumed in higher quantities (in grams) to be detected in urine, as only a small amount is excreted unchanged (Donoghue et al., 2000; Sharma et al., 2004). Previous studies' low sensitivity and higher detection limit with longer run time prove them inapplicable for measuring the CCM in plasma and urine samples.

In general, the bioactive components of spices, including turmeric, are not extensively bioavailable (Anand et al., 2007). The previously reported analytical methods for quantifying CCM in urine and plasma are not sensitive enough or use unpractical sample preparation procedures. For example, in an animal study, 0.1 g/kg of CCM oral administration resulted in only 2.25 μg/mL peak plasma concentration (Pan et al., 1999). Moreover, in rats with an oral dose of 500 mg/kg of CCM, a peak plasma concentration of 1.8 ng/mL was observed (Ireson et al., 2001). Different studies reported a wide range of LLOQ from 5 ng/mL to 11.8 μg/mL (Heath et al., 2003; Kim et al., 2017; Korany et al., 2017; Ma et al., 2015), or the lower plasma levels of CCM detected in cancer patients after consuming 8 g/day for 8 weeks were reported to be at 1.8 ng/mL level (Dhillon et al., 2008). In some other cases, the extraction and detection methods were unsuitable for quantifying the CCM as its concentrations were lower than other methods' limits of quantification (Kunati et al., 2018). The most sensitive method utilized in the phase I clinical trial of oral CCM had an LLOD of 1.84 ng/mL with a run time of 40 min. This method was sixfold less sensitive and had five times longer run time than the current work (Sharma et al., 2004). Kunati et al. reported concentrations ranging from 2.5 to 500 ng/mL, which precluded CCM detection in almost all tested samples, as they showed a CCM level was lower than the LLOQ (Kunati et al., 2018).

In the current study, detectable levels of CCM were measured in urine, a biofluid requiring a non‐invasive collection method, following oral consumption of a relatively moderate amount of turmeric. For human studies of health outcomes of habitual turmeric consumption, this new method offers significant benefits in terms of validity of results, lower cost, and higher participant acceptability of study activities. Additionally, objective approaches, such as the ones utilized in the current study, are not subject to the limitations of methods based on food intake analysis, including the unreliable nature of self‐reported food intake, imprecise and variable nutrient composition databases, and the inability to measure nutrient absorption rates (Picó et al., 2019). Collectively, this facilitates nutrient biomarker development, which is a priority for improving the accuracy of the nutritional status assessment.

The efficient method of quantifying CCM in urine presented here could offer valuable options in several scenarios: (1) Determining the absorption and bioavailability of CCM from specific foods/food combinations/supplements; (2) Establishing the correlation of CCM levels with biomarkers of health outcomes such as inflammatory cytokines; and (3) Characterizing intestinal microbiota profiles that are associated with higher or lower CCM levels to improve our understanding of the bidirectional relationship between microbiota communities and curcumin/turmeric bioactivity (He et al., 2015; Scazzocchio et al., 2020).

The application section of this study had some limitations due to the enrolment of a small number (*n* = 3) and gender composition (two males, one female) of subjects in the pilot clinical study. This issue affected the significance of the difference observed in pharmacokinetic parameters. However, due to the crossover nature of the study, each individual served as their own control, and the effect size was big enough that a strong trend was observed for the 24‐h urinary excreted amount of CCM (fourfold increase, *p* = .0776) and half‐life (twofold increase, *p* = .0527). Increasing the sample size can address that issue in larger studies. This preliminary study proves the current sensitive method's feasibility for quantifying low levels of CCM in biological matrix. Further studies with larger sample sizes utilizing this robust method could confirm our results and correlate CCM's beneficial effects with its plasma concentrations and total body exposure.

In conclusion, this study indicates that piperine significantly increased CCM oral absorption, reduced systemic clearance, and improved bioavailability as the 24‐h urinary extraction of CCM increased when it was used in combination with pepper. In addition, the current simple, selective, sensitive, and rapid LC–MS/MS method was developed and validated for simultaneous measurement of CCM and glibenclamide in human urine samples. This method is advantageous for the simple extraction procedure, higher sensitivity, and shorter run time, which can improve the validity and design of human nutrition studies about the effects of turmeric consumption on health.

## AUTHOR CONTRIBUTIONS

**Sana Khajeh pour:** Formal analysis (equal); methodology (equal); software (equal); validation (equal); visualization (equal); writing – original draft (lead). **Cynthia Blanton:** Conceptualization (equal); data curation (equal); formal analysis (equal); funding acquisition (lead); methodology (supporting); project administration (supporting); resources (supporting); writing – review and editing (supporting). **Biwash Ghimire:** Formal analysis (supporting); software (supporting); validation (supporting); writing – review and editing (supporting). **Ali Aghazadeh‐Habashi:** Conceptualization (equal); formal analysis (equal); investigation (lead); methodology (equal); project administration (lead); resources (lead); software (equal); supervision (lead); validation (equal); visualization (lead); writing – review and editing (lead).

## FUNDING INFORMATION

This research was funded by the Academy of Nutrition and Dietetics Foundation and the McCormick Science Institute Research. Additional funding was provided by the Academy of Nutrition and Dietetics Dietitians in Integrative and Functional Medicine Dietetic Practice Group.

## CONFLICT OF INTEREST STATEMENT

The authors declare no conflict of interest.

## INFORMED CONSENT STATEMENT

Local ethical committee approval has been received (protocol IRB‐FY2021‐7), and the informed consent of all participating subjects was obtained.

## Data Availability

The data that support the findings of this study are available on request from the corresponding author. The data are not publicly available due to privacy or ethical restrictions.
